# Divides Within the LGBT Community: Exploring the Impact of Generational Stereotypes

**DOI:** 10.1093/geroni/igaa057.1714

**Published:** 2020-12-16

**Authors:** Kinsey Bryant-Lees, Mary Kite

**Affiliations:** 1 Northern Kentucky University, Highland Heights, Kentucky, United States; 2 Ball State University, Muncie, Indiana, United States

## Abstract

Age is a unique, often overlooked, aspect of identity, which is particularly problematic within the LGBT community. While sexuality, sexual orientation, and gender identity are central to the identities of LGBT people, they are verboten for older adults. Thus, older LGBT individuals’ voices are inadvertently silenced. This talk will present data that demonstrates the reinforcing role that stereotypes play in maintaining the generational divide and address some of the unique inter-generational differences within the LGBT+ community that arise from differential experiences of cultural acceptance and historical events of LGBT youth coming of age across decades. For example, in the 1970’s the DSM criteria would have classified LGBT people as mentally ill; stark contrast to the 2010’s in which LGBT youth have grown up with legal protections against hate crimes, and marriage equality as a basic right. We will conclude with a discussion on cultivating community and productive conversations across generations.

